# 非小细胞肺癌新辅助免疫治疗进展

**DOI:** 10.3779/j.issn.1009-3419.2022.101.29

**Published:** 2022-07-20

**Authors:** 超 郭, 家齐 张, 单青 李

**Affiliations:** 100730 北京，中国医学科学院，北京协和医学院，北京协和医院胸外科 Department of Thoracic Surgery, Peking Union Medical College Hospital, Chinese Academy of Medical Sciences and Peking Union Medical College, Beijing 100730, China

**Keywords:** 肺肿瘤, 新辅助治疗, 免疫治疗, Lung neoplasms, Neoadjuvant therapy, Immunotherapy

## Abstract

非小细胞肺癌新辅助免疫治疗发展迅速，包括新辅助单药/双药免疫治疗、新辅助化疗/放疗联合免疫治疗等；部分临床试验结果显示，在经过选择的非小细胞肺癌患者中，新辅助免疫治疗具有可观的病理缓解率并产生可观察到的临床获益，而且毒副作用可耐受。Nivolumab联合化疗已于近期被美国食品药物监督管理局（Food and Drug Administration, FDA）批准用于非小细胞肺癌新辅助治疗。非小细胞肺癌新辅助免疫治疗的长期疗效、副作用以及手术相关问题需要进一步关注。

## Abstract (en)

Neoadjuvant immunotherapy, including neoadjuvant single- or dual-drug immunotherapy or combined immunotherapy with chemotherapy or radiotherapy, has witnessed a rapid development in non-small cell lung cancer. Clinical trials exhibited the encouraging pathological responses and certain clinical benefits in selected patients, with tolerable toxicity. Nivolumab with chemotherapy has been approved by Food and Drug Administration (FDA) as the first immunotherapy-based treatment for non-small cell lung cancer in the neoadjuvant treatment setting. There is the need for further evaluation of long-term efficacy, side effects or surgical issues for neoadjuvant immunotherapy in non-small cell lung cancer.

肺癌作为全球癌症相关死亡的首要原因，其治疗模式在近20年发展迅速，尤其对于非小细胞肺癌（non-small cell lung cancer, NSCLC）。肺癌免疫治疗的雏形可能是卡介苗的应用^[1, 2]^，其介导非特异性免疫反应抗肿瘤。免疫检查点的发现为肺癌特异性免疫治疗提供了更清晰的思路；免疫检查点抑制剂（immune checkpoint inhibitors, ICIs）通过结合肿瘤以及肿瘤微环境中的免疫检查点，阻断相应的信号通路，重新激活T细胞的抗肿瘤活性，抑制肿瘤细胞增殖。目前主要的免疫检查点包括细胞毒性T淋巴细胞相关抗原4（cytotoxic T lymphocyte-associated antigen 4, CTLA-4）、程序性死亡受体1（programmed cell death protein 1, PD-1）以及程序性死亡配体1（programmed cell death-ligand 1, PD-L1）；常见的ICIs为CTLA-4抗体（Ipilimumab、Tremelimumab）、PD-1抗体（Pembrolizumab、Nivolumab）和PD-L1抗体（Atezolizumab、Durvalumab）；除Tremelimumab外，其余5种ICIs均已获得美国食品药品监督管理局（Food and Drug Administration, FDA）批准用于一定适应证的NSCLC^[3]^。PD-1抗体（Pembrolizumab、Nivolumab）和PD-L1抗体（Atezolizumab、Durvalumab）也陆续在大陆获批了适应证；此外国产PD-1抗体（Sintilimab和Tislelizumab）已于国内获批不可切除的局部晚期或转移性NSCLC的一线治疗适应证。免疫治疗在晚期NSCLC上的应用经验，为相对早期的NSCLC提供了新的治疗策略。源于新辅助化疗的经验，新辅助治疗不仅能改善部分患者的生存^[4, 5]^，而且相比于辅助治疗，患者具有更好的耐受性^[6]^；另外，Nivolumab的研究^[7]^发现，术前Nivolumab诱导治疗后可导致T细胞克隆的扩增，这可能是新辅助免疫治疗（neoadjuvant immunotherapy, NAI）抗肿瘤优势之一。迄今，大量的临床试验探讨不同ICIs对于NSCLC新辅助治疗的有效性及安全性，本文综述相关进展。

## 1 新辅助治疗的指征及疗效评价

新辅助治疗一方面能评估药物的治疗反应，缩小原发灶，降低肿瘤期别，提高原发病灶的可切除性；另一方面还能够治疗微转移灶。美国国家综合癌症网络指南^[8]^推荐新辅助治疗的肿瘤主要为部分ⅡB期-Ⅲ期NSCLC，包括T3_*invasion*_N0-1M0，T4_*extension*_N0-1M0，T4N0-1M0及T1-3N2M0；而目前各临床试验、专家共识^[9]^普遍将NAI的适应人群扩展至ⅠB期-Ⅲ期NSCLC患者。国外绝大多数NAI临床试验采用第7版肺癌肿瘤原发灶-淋巴结-转移（tumor-node-metastasis, TNM）分期筛选试验人群，这导致部分患者的期别差异。此外，目前NAI临床试验大多将驱动基因阳性的患者排除在外，可能与这部分患者ICIs的疗效欠佳^[10]^有关。

新辅助治疗的短期疗效评价，包括影像学和病理学评估。1981年，世界卫生组织首先明确提出肿瘤治疗反应的规范定义，其中包括目前常用的客观缓解指标，即完全缓解、部分缓解、无变化和疾病进展等^[11]^。基于此，实体瘤疗效评价标准^[12]^、免疫相关的缓解标准^[13]^、实体瘤免疫疗效评价标准^[14]^等先后被提出并不断更新。解剖及功能的测量、病变数量及淋巴结的评估、影像学缓解指标的更新、正电子发射断层显像/计算机断层扫描（positron emission tomography/computed tomography, PET/CT）的评估、假进展、超进展以及总肿瘤负荷的提出是不同评估标准迭代更新的要点，但依然存在一些争议，这也是目前各临床研究的关注点。

国际肺癌研究协会基于肺癌病理缓解程度与患者生存期的相关性，于2020年对新辅助治疗后的肺癌病理评估作了明确定义^[15]^。主要病理缓解（major pathologic response, MPR）要求≤10%的残余肿瘤；病理完全缓解（pathologic complete response, pCR）指，肺癌所有切除标本，包括区域淋巴结，苏木精-伊红染色切片完全评估后无任何残余肿瘤细胞。IONESCO研究^[16]^是首个报道NAI后NSCLC病理缓解程度与总生存期（overall survival, OS）及无疾病生存期相关性的试验。作为目前大多数NAI临床试验的主要终点，病理缓解状态与患者长期生存结局的相关性仍需进一步验证。

## 2 ICIs的前瞻性新辅助治疗临床研究

### 2.1 Ipilimumab

2017年，Yi等^[17]^公布了TOP 1201研究的结果，其纳入ⅠB期-ⅢA期NSCLC，第1程为单纯新辅助化疗，第2-3程为化疗联合Ipilimumab；共24例患者入组，最终7例患者未手术，2例患者由于Ipilimumab相关的腹泻而延迟手术；58%的患者部分缓解，8%的患者疾病进展，其余稳定，中位OS为29.2个月（95%CI: 22.1-∞）。

NEOSTAR研究是比较Nivolumab单药和Nivolumab联合Ipilimumab的Ⅱ期临床试验^[18]^，其纳入Ⅰ期-ⅢA期NSCLC，术前接受3个周期Nivolumab或Nivolumab联合Ipilimumab（单次）治疗；共44例患者入组，39例患者完成手术，均为R0切除。Nivolumab单药组的MPR和pCR率分别为22%和9%，Nivolumab联合Ipilimumab组分别为38%和29%；截止数据发表时，其中位OS及肺癌相关的中位无复发生存期均未达到。NEOSTAR研究^[19]^的术后并发症与新辅助化疗后手术或直接手术相当。INCREASE研究^[20]^是一项在研的Ⅱ期临床试验，拟评估同步放化疗联合Nivolumab和Ipilimumab用于新辅助治疗cT3-4N0-1M0的NSCLC患者的有效性，结果待公布。

### 2.2 Nivolumab

单独针对Nivolumab的NAI临床试验主要有3项。2018年，一项单臂的Ⅱ期临床试验^[7]^纳入21例可切除的Ⅰ期-ⅢA期NSCLC患者，术前接受2程Nivolumab单药治疗，1例出现肿瘤无法切除，20例接受手术，9例出现MPR，8例出现降期；16例在术后12个月内无复发。

NADIM研究^[21]^是一项针对ⅢA期局部可切除NSCLC的Ⅱ期临床试验，患者术前采用3程Nivolumab联合化疗的新辅助方案，术后行Nivolumab辅助治疗直至12个月。截止报道时，一共46例患者入组，41例接受手术，34例出现MPR（包括26例pCR）；2年无进展生存率达77.1%。

CheckMate 816研究是迄今唯一有结果报道的Ⅲ期NAI临床试验^[22]^，其纳入可切除、ⅠB期-ⅢA期NSCLC，随机对照3程单纯化疗与化疗联合Nivolumab的新辅助疗效；术后行传统辅助治疗。化疗联合免疫新辅助治疗组（实验组）有83.2%患者完成手术，且83.2%的患者完成根治性切除；而单纯新辅助化疗组（对照组）仅75.4%患者完成手术，其根治性切除率约为77.8%；实验组中位无事件生存期（event-free survival, EFS）达31.6个月（95%CI: 30.2-未达到），而对照组为20.8个月（95%CI: 14.0-26.7）；实验组MPR和pCR率分别为36.9%和24.0%，对照组分别为8.9%和2.2%；同时研究认为ⅢA期患者比ⅠB期、Ⅱ期患者更能获益。Nivolumab成为首个获得批准用于NSCLC新辅助治疗的ICI^[23]^。

### 2.3 Pembrolizumab

Eichhorn等^[24]^于2019年公布了Pembrolizumab的NEOMUN研究的方案，其纳入Ⅱ期-ⅢA期可切除的NSCLC，术前接受2程的Pembrolizumab诱导治疗，术后进行辅助化疗和/或放疗。NEOMUN研究^[25]^初步入组15例患者，所有患者接受R0切除；3例患者由于治疗相关副作用（treatment-related adverse event, TRAE）等偏离了研究方案。分别有2例患者达到MPR和pCR。Tong等^[26]^近期公布了TOP1501研究的Ⅱ期试验结果，ⅠB期-ⅢA期的NSCLC患者术前接受2程的Pembrolizumab新辅助治疗，术后接受4程的Pembrolizumab辅助治疗；共30例患者入组，最终5例患者由于疾病进展不适合手术；11例患者出现降期，22例患者达到R0切除，80%的肿瘤病理缓解超过50%，28%的患者出现MPR，12%的患者出现pCR。

### 2.4 Atezolizumab

2018年，美国临床肿瘤协会年会以摘要形式首次公布了Atezolizumab的Ⅱ期临床试验的初步结果^[27]^，入组的ⅠB期-ⅢB期、可切除NSCLC患者，术前接受2程Atezolizumab诱导治疗；摘要公布了前21例患者结局，1例患者出现中枢神经系统转移，1例肿瘤无法切除，4例患者出现MPR，11例患者病理缓解≥50%；目前未见最终结果报道。

2020年，首项关于NSCLC化疗联合PD-L1抗体的新辅助治疗Ⅱ期临床试验^[28]^结果公布，其纳入ⅠB期-ⅢA期可切除NSCLC，术前接受4程化疗联合Atezolizumab诱导治疗；术后无其他系统性治疗；最终入组30例患者，29例患者接受手术，26例患者完成R0切除；试验过程中未观察到新辅助治疗相关的手术并发症。

### 2.5 Durvalumab

目前在研的关于Durvalumab的Ⅲ期、双盲、安慰剂对照的临床试验（AEGEAN研究）^[29]^，拟入组可切除的Ⅱ期-Ⅲ期NSCLC，术前采用化疗联合Durvalumab/安慰剂，术后辅助Durvalumab/安慰剂，拟评估Durvalumab是否可以改善病理及临床结局，尚无结果公布。

2021年，瑞士临床癌症研究小组公布了一项多中心、单臂的Ⅱ期临床试验^[30]^结果，其纳入经病理证实的T1-3N2M0的NSCLC，患者先接受标准的3程新辅助化疗，后序贯2程Durvalumab诱导治疗；术后维持Durvalumab治疗1年；共纳入67例患者，55例完成手术，51例为R0切除；62%的患者达到MPR（18%为pCR）。

2021年，Altorki等^[31]^首次公布ICIs联合放疗的新辅助治疗的Ⅱ期试验结果，其纳入Ⅰ期-ⅢA期、可切除的NSCLC，比较新辅助Durvalumab单药和Durvalumab联合立体定向放疗的效果；所有患者接受2程Durvalumab治疗，联合治疗组在第一程Durvalumab给药前连续3 d接受每天一次8 Gy的放疗；术后接受传统的辅助治疗，亦可考虑每月一次的Durvalumab治疗持续12个月。共60例患者入组，每组30例；52例（每组各26例）患者接受手术。Durvalumab单药新辅助组，2例患者出现MPR；Durvalumab联合放疗组，16例患者出现MPR。此外，SQUAT研究（WJOG 12119L）^[32]^是一项针对ⅢA期/ⅢB期NSCLC、新辅助同步化疗-免疫-放疗、术后序贯辅助免疫治疗的Durvalumab的Ⅱ期临床试验，结果尚未公布。

### 2.6 Sintilimab

中国医学科学院肿瘤医院高树庚教授^[33]^首先于2020年报道了Sintilimab的ⅠB期临床试验结果；入组的IA期-ⅢB期NSCLC患者于术前接受2程Sintilimab治疗，术后接受传统化疗、Sintilimab或者化疗联合Sintilimab辅助治疗；一共纳入40例患者，37例完成手术，36例手术为R0切除，2例患者由于TRAE导致治疗延迟；最终15例达MPR，6例达pCR，但均为鳞癌患者。吉林大学第一医院的Sun等^[34]^近期公布了Sintilimab在潜在可切除的ⅢA期/ⅢB期NSCLC患者中新辅助治疗的Ⅱ期临床试验结果，所有患者术前接受2程-3程化疗联合Sintilimab治疗，术后接受辅助治疗；共20例患者入组，16例完成手术，均为R0切除；10例达到MPR，其中5例为pCR（均为鳞癌）。上海肺科医院的Zhang等^[35]^报道了50例ⅢA期NSCLC患者使用化疗联合Sintilimab新辅助治疗的结果，一共50例患者入组，2程-4程新辅助治疗后，23例部分缓解，24例疾病稳定，30例患者接受手术（均为R0切除），13例达MPR（包括6例pCR）。

### 2.7 Toripalimab

尽管Toripalimab暂无NSCLC的适应证，但NAI的临床试验已有报道。一项关于Toripalimab应用于可切除、ⅢA期或T3-4N2 ⅢB期NSCLC的Ⅱ期临床试验^[36]^，设计术前3程化疗联合Toripalimab的治疗；该研究入组33例患者，30例患者接受手术，29例为R0切除；20例患者达MPR，其中15例为pCR，24例患者达到降期。

除此之外，国内尚有数项已开展的针对多种ICIs的临床研究^[37, 38]^。NSCLC的NAI临床试验归纳于表 1；需指出的是，部分临床试验pCR的计算未包含淋巴结的病理评估。此外，已有报道将NAI用于晚期、不可切除的NSCLC患者人群^[39-41]^，亦能实现一定的肿瘤可切除性及生存获益。一项纳入16项研究的*meta*分析^[42]^显示，NAI后综合可切除率85.8%，MPR率可达43.5%，pCR率约21.9%；新辅助化疗联合免疫治疗总体MPR率可达53.3%，pCR率约28.6%，较单药NAI组显著升高；总体而言，包含ICIs的新辅助治疗能够实现较高的病理缓解，类似的结论被多个*meta*分析所证实^[43, 44]^。但病理缓解状态以及短期内EFS的延长，能否转化为OS的获益，尚存争议。

**表 1:** NSCLC新辅助免疫治疗的临床试验汇总 Clinical trials for neoadjuvant immunotherapy in NSCLC

Study	Phase	Stage	Cases of NAI	Intervention	NAI cycle	R0 resection	MPR^a^	pCR^a^	Survival	Note
Forde^[22]^	3	ⅠB-ⅢA NSCLC	179	Nivolumab+ chemotherapy *vs* chemotherapy	3	83.2% *vs* 77.8%	36.9% *vs* 8.9%	24.0% *vs* 2.2%	EFS: 31.6 mon *vs* 20.8 mon	Non-squamous type showed greater benefit
Forde^[7]^	2	Ⅰ-ⅢA NSCLC	21	Nivolumab alone	2	95.2%	45.0%	15.0%	18 mon RFS: 73%	A higher mean mutational burden was associated with MPR
Provencio^[21]^	2	ⅢA NSCLC	46	Nivolumab+ chemotherapy	3	89.1%	82.9%	63.4%	12 mon PFS: 100% 18 mon PFS: 91.9% 24 mon PFS: 87.9%	No significant difference in PFS between incomplete pathological response and MPR
Yi^[17]^	2	ⅠB-ⅢA NSCLC	24	Chemotherapy+ phased in Ipilimumab	1+2	54.2%	NA	15.0%^[45]^	Median OS: 29.2 mon	Ipilimumab had little to no effect on circulating Tregs and myeloid-derived suppressor cells
Cascone^[18]^	2	Ⅰ-ⅢA NSCLC	44 (23 *vs* 21)	Nivolumab *vs* Nivolumab+ Ipilimumab	3	91.3% *vs* 76.2%	24.0% *vs* 50.0%	10.0% *vs* 38.0%	NA	Tumor specimens after Nivolumab+Ipilimumab had fewer viable tumor cells
Reuss^[46]^	1b/2	ⅠB-ⅢA NSCLC	9	Nivolumab+ Ipilimumab	3	67.0%	NA	33.0%	NA	Pathologic response was significantly associated with pre-treatment tumor PD-L1 expression
Rusch^[27]^	2	ⅠB-ⅢB NSCLC	21	Atezolizumab alone	2	90.5%	21.0%	NA	NA	Adjuvant therapy consisted of up to 12 months of atezolizumab
Shu^[28]^	2	ⅠB-ⅢA NSCLC	30	Atezolizumab+ chemotherapy	4	86.0%	57.0%	33.0%	DFS: (Non-MPR *vs* MPR) 14.3 mon *vs* 34.5 mon	The first published trial of chemotherapy plus PD-L1 inhibitor in the neoadjuvant setting
Rothschild^[30]^	2	T1-3 N2M0 NSCLC	67	Chemotherapy+ sequential Durvalumab	3+2	76.0%	62.0%	18.0%	EFS: 1-year 73% 2-year 68%	Adjuvant Durvalumab given every two weeks for 26 cycles
Altorki^[31]^	2	Ⅰ-ⅢA NSCLC	30 *vs* 30	Durvalumab *vs* Durvalumab plus stereotactic radiotherapy	2	77.0% *vs* 83.0%	7.7% *vs* 61.5%	0.0% *vs* 30.8%	NA	All the patients were offered adjuvant chemotherapy or radiotherapy, or both, or durvalumab No consistent effect of radiation on PD-L1 expression in tumor samples
Eichhorn^[25]^	2	Ⅱ-ⅢA NSCLC	15	Pembrolizumab alone	2	100.0%	13.3%	13.3%	NA	Patients with PD-L1 expression of ≥10% were more likely to be at least MPR
Tong^[26]^	2	ⅠB-ⅢA NSCLC	30	Pembrolizumab alone	2	73.3%	80.0%	12.0%	NA	Patients received 4 cycles of adjuvant pembrolizumab after completion of standard adjuvant therapy
Gao^[33]^	1b	ⅠA-ⅢB NSCLC^b^	40	Sintilimab alone	2	90.0%	40.5%	16.2%	NA	MPR/pCR were all squamous NSCLC
Sun^[34]^	2	ⅢA/B NSCLC^b^	20	Sintilimab+ chemotherapy	2-3	80.0%	62.5%	31.3%	NA	Adjuvant sintilimab+chemotherapy 2 cycle commence 28 days-60 days after surgery
Zhang^[35]^	2	ⅢA NSCLC	50	Sintilimab+ chemotherapy	2-4	60.0%	43.3%	20.0%	12 mon DFS: 85.3% 12 mon OS: 93.7%	FEV_1_ and FEV_1_% predicted improved significantly after neoadjuvant therapy
Zhao^[36]^	2	ⅢA/B NSCLC^b^	33	Toripalimab+ chemotherapy	3	87.9%	66.7%	50.0%	NA	Adjuvant toripalimab commencing 4 weeks-8 weeks after surgery until 12 months
AE: adverse event; DFS: disease-free survival; EFS: event-free survival; MPR: major pathologic response; mon: month; NA: not available; NSCLC: non-small cell lung cancer; pCR: pathologic complete response; PFS: progression-free survival; RFS: recurrence-free survival; *vs*: versus; PD-L1: programmed cell death-ligand 1; FEV_1_: Forced expiratory volume in one second ^a^ The rates of MPR and pCR were the percentage of patients with surgery, the rate of R0 resection was the percentage of patients enrolled. ^b^TNM staging classification was referred to the 8^th^ edition.										

## 3 新辅助免疫治疗与手术

### 3.1 新辅助治疗-手术时间窗

新辅助治疗除了潜在的生存获益，亦存在相应的风险，包括药物直接毒副作用、后续治疗的中断甚至增加手术并发症的发生率等。因此，新辅助治疗与手术之间通常存在一定的间期；一方面，药物治疗后肿瘤的反应需要一定的时间窗口；另一方面，新辅助治疗的毒副作用同样需要时间缓解，以利于患者接受后续治疗；甚至新辅助治疗后不同时间点的手术可能会遇到手术部位组织条件的差异性表现。2016年，Gao等^[47]^通过回顾分析美国国家癌症数据库1, 623例ⅢA期NSCLC患者新辅助同步放化疗的资料时，将新辅助治疗结束-手术的间期分为0周-3周、3周-6周、6周-9周及9周-12周（超过12周者被排除在外）；发现间期在6周以内手术的患者无显著生存差异，而6周-9周及9周-12周手术的患者OS显著下降。2020年，Rice等^[48]^对同一数据库后期5, 946例ⅢA期NSCLC患者进行分析，患者接受新辅助放化疗、化疗或者放疗，其根据所有新辅助治疗结束-手术间期的四分位距，将患者分为三组，间期 < 77 d、77 d-114 d及 > 114 d；发现手术间期并不影响早期死亡率，但间期较短者1年及3年生存期更长。两项研究均为回顾性分析，故而缺乏手术并发症的信息。既往针对直肠癌的回顾性数据分析^[49]^显示，相比于新辅助放化疗与手术间期 > 10周的患者，10周以内手术的患者具有更高的切口感染率。以上研究均不包含NAI。表 2总结了不同临床研究的计划手术时间点；大多临床试验设计患者手术时间点在NAI结束后的3周-6周，TOP 1501研究^[26]^要求手术在新辅助治疗后至少2 d后，TOP 1201研究^[45]^设计手术在新辅助治疗结束后12周内。但理想的NAI与手术的间期究竟应该多久，目前尚无法回答。

**表 2:** NSCLC新辅助免疫治疗手术时间点、毒副作用及并发症 Surgery timing, adverse events and complications in clinical trials for neoadjuvant immunotherapy in NSCLC

Study	Stage	Intervention	Scheduled surgery timing	≥3 grade TRAE	Surgical complication	≥3 grade postoperative AE
Forde^[22]^	ⅠB-ⅢA NSCLC	Nivolumab+ chemotherapy *vs* chemotherapy	Within 6 weeks after the completion of neoadjuvant treatment	33.5% *vs* 36.9%	41.6% *vs* 46.7%	11.4% *vs* 14.8%
Forde^[7]^	Ⅰ-ⅢA NSCLC	Nivolumab alone	About 4 weeks after the first dose	4.5%	NA	NA
Provencio^[21]^	ⅢA NSCLC	Nivolumab+ chemotherapy	42 days-49 days after the first day of the third cycle	30.0%	29.0%	NA
Yang^[45]^	Ⅱ-ⅢA NSCLC	Chemotherapy+ phased in Ipilimumab	Within 12 weeks after completion of neoadjuvant therapy	46.0%	69.2%	NA
Cascone^[18]^	Ⅰ-ⅢA NSCLC	Nivolumab *vs* Nivolumab+ Ipilimumab	Between 21 days and 42 days after the last dose of nivolumab	13.0% *vs* 10.0%	38.0% *vs* 31.0%^[19]^	NA
Reuss^[46]^	ⅠB-ⅢA NSCLC	Nivolumab+ Ipilimumab	6 weeks after the first dose	33.0%	< 22.0%^a^	< 22.0%^a^
Rusch^[27]^	ⅠB-ⅢB NSCLC	Atezolizumab alone	Day 40±10 after the first dose	4.8%	NA	NA
Shu^[28]^	ⅠB-ⅢA NSCLC	Atezolizumab+ chemotherapy	Approximately 4 weeks after the last dose of treatment	91.0%^a^	NA	NA
Besse^[53]^	IA (≥2 cm)- ⅢA non N2 NSCLC	Atezolizumab alone	Between day 21-28	0.0%	10.3%	3.4%
Rothschild^[30]^	T1-3N2M0 NSCLC	Chemotherapy+ sequential Durvalumab	2 weeks-4 weeks after the last application of durvalumab	88.0%	NA	NA
Altorki^[31]^	Ⅰ-ⅢA NSCLC	Durvalumab *vs* Durvalumab plus radiotherapy	Within 2 weeks-6 weeks following the second cycle of durvalumab	17.0% *vs* 20.0%	NA	38.5% *vs* 38.5%
Eichhorn^[25]^	Ⅱ-ⅢA NSCLC	Pembrolizumab alone	Between day 43-50	20.0%	7.0%^a^	NA
Tong^[26]^	ⅠB-ⅢA NSCLC	Pembrolizumab alone	At least 2 days after the second dose of pembrolizumab	3.3%	48.0%	NA
Gao^[33]^	IA-ⅢB NSCLC	Sintilimab alone	Within 29 days to 43 days after the first dose of sintilimab	10.0%	10.0%	NA
Sun^[34]^	ⅢA/B NSCLC	Sintilimab+ chemotherapy	30 days-45 days after neoadjuvant treatment	35.0%	> 20.0%^a^	20.0%
Zhang^[35]^	ⅢA NSCLC	Sintilimab+ chemotherapy	Within 42 days to 49 days after the last dose of chemotherapy	8.0%	NA	NA
Zhao^[36]^	ⅢA/B NSCLC	Toripalimab+ chemotherapy	4 weeks-5 weeks following the first day of the third cycle	NA	NA	NA
TRAE: treatment-related adverse ^a^The data was generated indirectly from the published articles.						

### 3.2 新辅助治疗术后并发症

新辅助治疗临床试验的围术期并发症报道相对隐晦。CheckMate 816研究^[22]^显示，新辅助化疗联合Nivolumab组TRAE发生率为33.5%，而新辅助化疗组为36.9%；两组分别有15.6%和20.7%的患者无法手术；结论认为，相比于单纯化疗，化疗联合Nivolumab不增加AE事件的发生率，不影响手术的可行性；但补充资料显示，新辅助Nivolumab联合化疗组具有更多严重的AEs、更多的治疗中断事件以及手术切口并发症。Durvalumab单药的NAI试验^[30]^报道了88%的患者出现了3级以上的AEs，包括2例致死性AEs；但Durvalumab联合放疗的新辅助治疗并不增加3级以上术后并发症发生率^[31]^。Sintilimab的ⅠB期临床试验^[33]^报道了AEs的发生率为80%，新辅助TRAEs发生率为52.5%。表 2列举了部分临床试验的并发症报道结果。手术并发症，尤其3级以上者少见文献直接报道。2016年，Li等^[50]^曾报道了针对30项肺癌新辅助治疗（不包含NAI）研究的*meta*分析，结果显示，新辅助治疗能够明显增加肺切除术后胸膜支气管瘘的发生率（OR=2.166, 95%CI: 1.398-3.357, *P*=0.001）。Sintilimab的Ⅱ期临床试验^[34]^中发现4例患者（共20例）出现胸膜支气管瘘，其中1例因此死亡；胸膜支气管瘘均发生在术后辅助治疗开始后，但同期其他Ⅲ期肺癌手术患者未发生胸膜支气管瘘；研究者认为部分患者新辅助治疗后的手术难度有所增加。Zhang等^[51]^则提出，Sintilimab新辅助治疗能增加并发症发生率，但并不增加手术难度。Jiang等^[52]^在一项回顾性的研究中报道了NAI后微创转开胸手术发生率约为3.2%。*Meta*分析^[43]^显示，相比于单纯NAI，新辅助化疗联合免疫治疗具有更高的TRAEs及更高的术后并发症发生率。不同研究存在一定的偏倚，正确识别、筛选出高风险人群，对于规避相应的严重事件至关重要。

## 4 新辅助免疫治疗术后辅助治疗的策略

目前，新辅助治疗后的辅助治疗策略需要多学科共同探讨，根据患者病理结果、身体状态、新辅助治疗方案等协商决定。部分临床试验设计了术后辅助治疗策略，目前主要分为两种，一种为传统的单独放疗、单独化疗或联合放化疗；另一种为包含ICIs的维持或者联合维持治疗，诸如Pembrolizumab（Tong^[26]^）、Atezolizumab（Rusch^[27]^）、Durvalumab（Rothschild^[30]^和Altorki^[31]^）以及Sintilimab（Sun^[34]^）；但方案的选择依据以及对患者生存的影响未见披露。虽然目前驱动基因阳性的患者被排除在多数NAI临床试验之外，但这部分患者的新辅助治疗后的辅助治疗策略亦需进一步探讨。

## 5 新辅助免疫治疗的优势人群筛选、预测

CheckMate 816研究^[22]^显示，化疗联合Nivolumab的新辅助治疗能够实现56%的循环肿瘤DNA（circulating tumor DNA, ctDNA）清除，而单纯的化疗新辅助仅实现35%的ctDNA清除，ctDNA清除率与EFS及pCR率相关；虽然在新辅助化疗联合Nivolumab组患者具有更多的Ⅲ期患者。Yue等^[54]^分析了一项纳入22例新辅助治疗的NSCLC患者的研究，结果认为与传统的CT相比，ctDNA与病理缓解具有更好的相关性，而且与新辅助化疗或新辅助双免疫治疗相比，新辅助化疗联合免疫治疗具有更好的ctDNA反应。目前，基于ctDNA的微小残留病变（minimal residual disease, MRD）的研究十分火热，但尚缺乏公认的、规范的MRD检测手段，以及高质量的证据验证MRD与患者预后的相关性，临床上MRD指导后续治疗的建议亦存在空白。

作为免疫检查点，PD-L1的表达水平亦被证明与患者ICIs疗效具有一定的相关性。一项*meta*分析^[55]^显示，与PD-L1表达 < 1%的患者相比，PD-L1表达≥1%的患者具有更高的MPR及pCR率；50%的PD-L1表达临界值对于预测MPR具有更好的有效性；同时高肿瘤突变负荷亦与MPR及pCR相关。

目前，PET/CT是临床上广泛使用的一种癌症影像评估工具。Sintilimab的Ⅰ期临床研究^[33]^发现，新辅助治疗前后PET/CT的最大标准摄取值（maximum standardized uptake value, SUVmax）衰减量与肺癌标本病理反应具有显著相关性。Eichhorn等^[25]^关于Pembrolizumab的研究明确指出，SUVmax下降≥25%者出现MPR及pCR的比例明显升高。Zhao等^[36]^关于Toripalimab的研究表明，在MPR及pCR组，患者的SUVmax下降更显著；另外，PET能够预测56.7%的原发肿瘤灶和53.3%的淋巴结病理缓解状态。

Laza Briviesca等^[56]^对NADIM研究中29例ⅢA期NSCLC患者诊断时以及新辅助治疗后的血液样本进行了分析，发现了外周血的免疫细胞亚型与患者pCR相关。Wang等^[57]^发现Sintilimab治疗后CD8^+^ PD-1^-^ T细胞与MPR具有临床相关性。免疫细胞亚型可能参与ICIs的作用机制，同时也可能是治疗的结局，其对治疗反应的预测有待验证。此外，Forde等^[7]^于2018年报道的关于Nivolumab的Ⅱ期临床试验中，12例治疗前的NSCLC组织进行了全外显子测序，11例接受手术治疗，病理缓解评估发现MPR组具有更高的基因突变负荷；突变负荷是否能预测NAI疗效尚需进一步探讨。

## 6 总结

ICIs新辅助治疗可切除NSCLC具有相对可观的病理缓解率，部分临床试验证实了一定的临床获益，毒副作用可控。患者的长期生存、严重的AEs事件以及手术相关事件需要我们更进一步的关注。有效识别获益人群，规避高风险事件，真正实现患者的高质量生存。
